# Effects of Assault Type on Cognitive Behaviour Therapy for Coexisting Depression and Alcohol Misuse

**DOI:** 10.3390/jcm6070072

**Published:** 2017-07-21

**Authors:** Kylie A. Bailey, Amanda L. Baker, Patrick McElduff, Mark A. Jones, Christopher Oldmeadow, David J. Kavanagh

**Affiliations:** 1 School of Medicine and Public Health, University of Newcastle, University Drive, Callaghan NSW 2308, Australia; amanda.baker@newcastle.edu.au (A.L.B.); patrick.mcelduff@newcastle.edu.au (P.M.); christopher.oldmeadow@hmri.org.au (C.O.); 2 Hunter Medical Research Institute, 1/1 Kookaburra circuit, New Lambton Heights NSW 2305, Australia; mark.jones@hmri.org.au; 3 Centre for Children’s Health Research, Institute of Health & Biomedical Innovation and School of Psychology & Counselling, Queensland University of Technology, GPO Box 2434, Brisbane QLD 4000, Australia; david.kavanagh@qut.edu.au

**Keywords:** violence, depression, alcohol drinking, cognitive behaviour therapy, treatment

## Abstract

Although assault exposure is common in mental health and substance misusing populations, screening for assaults in treatment settings is frequently overlooked. This secondary analysis explored the effects of past sexual (SA) and physical (PA) assault on depression, alcohol misuse, global functioning and attrition in the Depression and Alcohol Integrated and Single focussed Intervention (DAISI) project, whose participants (N = 278) received cognitive behaviour therapy (CBT) for their depression and/or alcohol misuse. Of the 278 DAISI participants, 220 consented to screening for past assault (either by a stranger or non-stranger) at baseline. Depression, alcohol, and global functioning assessments were administered at baseline and 3, 12, 24, and 36 months post baseline. A between-group analysis was used to assess differences between SA and No SA, and PA and No PA groupings, on adjusted mean treatment outcomes across all assessment periods. SA and PA participants had similar mean symptom reductions compared to No SA and No PA participants except for lower depression and global functioning change scores at the 12-month follow-up. People with coexisting depression and alcohol misuse reporting SA or PA can respond well to CBT for depression and alcohol misuse. However, follow-up is recommended in order to monitor fluctuations in outcomes.

## 1. Introduction

Assault exposure across the lifetime is often reported in mental health [1] and substance/alcohol misuse populations [2,3]. Victim of crime rates (including physical (PA) and sexual assaults (SA)) in mental health populations are 11 times higher than the general community, especially for those with a severe mental illness [4]. Rates of assault (across the life time) are also high in substance misuse populations [5,6] with 90% reporting exposure to multiple traumas [3,7]. Research has also found that people who report exposure to intentional traumas (such as sexual and physical assault) are also more likely to experience comorbid posttraumatic stress symptoms (PTSS), alcohol misuse, and depression, compared to those who experience non-intentional traumas [8,9,10]. High rates of PTSS, alcohol misuse, and depression are also reported in people who experienced childhood abuse [2,11].

Studies investigating treatment outcomes following an assault have found posttraumatic stress disorder (PTSD)-focused Cognitive Behaviour Therapies (CBT) effective in reducing assault-related PTSS [12,13] and depressive symptoms [14,15,16]. The PTSD-focused CBT model has also been found effective in treating PTSD that coexists in substance/alcohol misuse populations [12,15,17,18]. However, most PTSD-CBT studies are limited in that they predominantly recruit female participants [14,19,20] while other studies show that relapse rates are higher in participants with coexisting PTSD and alcohol misuse [5,21]. Furthermore, studies on treatment outcomes in participants with coexisting history of exposure to intentional traumas (such as interpersonal violence) and alcohol misuse, tend to have much poorer symptom improvement compared to those without these experiences [5].

Despite established high prevalence rates of assault across the lifetime in mental health populations [22] and in coexisting depressed and alcohol misuse populations [6,23], screening for traumatic events tends not to occur in health services [22]. If exposure to traumatic events is not assessed in health settings, then the CBT treatments that are provided may not include the necessary PTSD-focused interventions. When trauma symptoms are not addressed (i.e., where participants receive non-PTSD focused CBT), those with comorbid depression [24] or alcohol misuse [25] have poorer treatment outcomes and higher recidivism rates compared to those without trauma symptoms.

This study is a secondary analysis of the Depression and Alcohol Integrated and Single focused Intervention (DAISI) project (parent study) [26,27], whereby we investigated how lifetime SA and/or PA effects treatment outcomes of people seeking depression and alcohol CBT. At the time of writing, no research into the effects of receiving non-PTSD focused CBT for coexisting depression and alcohol misuse in this treatment seeking population, who have also experienced traumatic assault (by a stranger and/or non-stranger) could be identified. Also unknown is how different assault types affect depression and alcohol treatment outcomes. Therefore, we aimed to explore the effects of past exposure to SA and PA on comorbid depression and alcohol misuse; and other outcomes along with rates of attrition, following CBT treatment for coexisting depression and alcohol misuse.

We predicted that participants who reported past SA or PA would show poorer treatment outcomes across follow-up, including more severe depressive symptoms, alcohol misuse (greater alcohol consumption and more severe dependence symptoms), and poorer global functioning compared to those without assault exposure. We also predicted that participants who reported past SA or PA would show poorer attendance during the treatment and follow-up assessments compared with those without assault exposure.

## 2. Methods

### 2.1. The Parent Study

The parent study was the DAISI project, which was a randomised controlled trial in Newcastle and Brisbane, Australia [26,27]. The DAISI project recruited participants seeking treatment for depression and alcohol misuse. Participants were randomly allocated to an initial single-session integrated intervention, or to one of three CBT interventions of nine additional sessions focusing on alcohol only, depression only, or alcohol and depression. The DAISI trial found that: longer interventions tended to be more effective in improving depression and functioning in the long term, and in reducing alcohol consumption in the short term. Integrated treatment was at least as effective as single-focused CBT. Alcohol-focused treatment was as effective as depression-focused treatment in reducing depression and more effective in reducing alcohol consumption. The best approach appeared to be an initial focus on both conditions (as per the initial session) followed by additional integrated- or alcohol-focused sessions [26,27].

### 2.2. Participants

Participants (*n* = 220) into this sub-study were recruited from the DAISI project (N = 278), with permission for this sub-analysis granted by the Human Research Ethics Committees of the University of Newcastle and the University of Queensland [27]. The inclusion criteria for the DAISI project were: (a) ≥16 years of age; (b) current depressive symptoms; (c) and consuming alcohol at harmful levels as determined by 2001 National Health and Medical Research Council (NHMRC) drinking guidelines [28]. Potential participants were excluded if they: (i) were currently diagnosed with a psychotic disorder; (ii) reported traumatic brain injury; (iii) lacked fluency in English; or (iv) lived too far away to attend sessions. Participants may have also concurrently received treatment for depression or alcohol misuse from other services.

### 2.3. Measures

Traumatic assault and PTSS were measured using the Posttraumatic Stress Diagnostic Scale (PDS) [29]. An assault was considered traumatic if the participant felt helpless or hopeless (Criterion A) during exposure [30]. PTSS severity was measured by summing questions 22–38 which were ranked from “5 or more times a week” (3), “2 to 4 times a week” (2), “once a week or less” (1), and “not at all or only once” (0). The score for PTSS severity ranges between 0 and 51 [29]. The PDS trauma checklist groups SA events into 3 event types: SA by a stranger; a non-stranger; or when under 18 by someone 5 years older than them. PA events types are grouped into PA by a stranger or a non-stranger [29].

Depressive symptoms for the 2 weeks before baseline were measured using the 21-item Beck Depression Inventory (BDI-II) [31]. Participants rated the severity of 21 symptoms of depression from 0 to 3, where 3 indicates highest severity. Questions 16 and 18 have seven options around changes in appetite and sleep. BDI scores range from 0 to 63 [31].

Alcohol misuse across the previous 6 months was measured with the Alcohol Use Disorders Identification Test (AUDIT) [32]. The AUDIT has 10 items with a score range between 0 and 36 with questions 1–3 assessing for levels of alcohol consumption, questions 4–6 assessing alcohol dependence symptoms, and questions 7–10 assessing for harms associated with alcohol use [32].

Symptoms of alcohol dependence within the 6-month period before baseline were assessed by the Severity of Alcohol Dependence Questionnaire (SADQ-C) [32]. Items 1–16 are scored as “nearly always” (3), “often” (2), “sometimes” (1), and “almost never” (0). The final four items (17–20) assess for physical withdrawal the morning after two days of heavy drinking, using a four point scale of “quite a lot” (3), “moderately” (2), “slightly” (1), and “not at all” (0). The score ranges between 0–60 [32]. The questionnaire has sound reliability and validity [32].

Drinking frequency was assessed by the Alcohol Timeline Followback (TLFB) method, focusing on the 2 weeks before baseline [33]. TLFB is a calendar method that accurately and retrospectively measures daily alcohol consumption (and the variability in consumption levels) [33].

Global functioning levels were determined by the Global Assessment of Functioning (GAF) [34] with scores ranging from 0 to 100.

### 2.4. Procedure

In the parent study, participants received AUD $20 as reimbursement for travel costs at the baseline and follow-up assessments. Treatment randomisation occurred following the brief integrated intervention session and was stratified by study site, gender and concurrent antidepressant or anti-craving medication. Independent psychologists (blind to treatment allocation) conducted all follow-up assessments.

### 2.5. Interventions

1. All participants received a single 90-minute session that focused on links between depression and alcohol misuse and included baseline assessment feedback, psychoeducation, and motivational interviewing. After the initial session, all participants were then randomly allocated to one of the four intervention streams: Brief intervention (no further treatment); or a further nine 1-hour sessions of:
2. The alcohol-focused CBT intervention;
3. The depression-focused CBT intervention; or
4. The integrated CBT intervention (combined depression and alcohol).

All treatments were fully manualized. If allocated to further treatment, the subsequent nine weekly 1-hour sessions were similarly structured and comprised of further motivational interviewing, mindfulness exercises, and CBT. Sessions 2–4 had a behavioural and skills training focus with participants learning to: monitor mood and/or cravings; develop change plans, and manage impulsive thoughts and/or cravings. Activity lists and scheduling were developed and mindfulness tasks commenced. Sessions 5–7 focused on identifying and managing unhelpful automatic thoughts, improving problem solving, and examining evidence for problematic schema and core beliefs. Sessions 8–10 were based on developing an emergency plan, practicing assertiveness or alcohol refusal skills, and relapse prevention techniques [35]. Integrated sessions addressed both depression and alcohol use as well as explaining how these conditions impacted upon each other.

### 2.6. Statistical Analysis

Due to the limited sample size, the analysis focused on the assault groupings of SA (non-stranger, stranger, and sexual contact <18 years) versus No SA, and PA (physical assault by a stranger and/or non-stranger) versus No PA.

Chi-square (χ^2^) tests of independence were performed on SA versus No SA group membership versus treatment allocation, gender, and current prescription of antidepressant medication. We conducted *t*-tests on SA versus No SA, and PA versus No PA at baseline for treatment attendance, days in treatment, PTSS, depression, alcohol consumption/misuse/dependence, and global functioning. To explore attrition, we used discrete-time survival analysis to obtain hazard ratios for SA versus No SA, and PA versus No PA, over the five follow-up periods. Participant characteristics were reported in percentages, means, and standard deviations.

Group effects for SA versus No SA, and PA versus No PA at the 3, 12, 24, and 36-month post-baseline assessments were assessed via linear mixed-effects regression on mean changes in symptom levels for the following treatment outcome variables: PTSS severity, depressive symptom; levels of alcohol consumption, severity of alcohol problems; severity of alcohol dependence symptoms; and global functioning. The analyses were adjusted for baseline symptoms, gender, antidepressant medication, and days in treatment. Analyses using the above treatment outcome variables were based on the intention-to-treat principle, whereby all participants are included in the analysis, regardless of treatment retention/attrition [36].

In supplementary analyses, we assessed (via linear mixed-effects regressions) the interaction effects of follow-up periods by SA status by PA status (and all lower order interactions) on the estimated mean changes from baseline in symptom levels. At each follow-up period, we compared the outcome measures for depression, global functioning, and alcohol dependence symptoms for the participants that had experienced both SA and PA with those participants experiencing neither SA nor PA, with those participants experiencing SA and No PA and, finally, with those participants experiencing *No SA* and *PA*. All models included adjustments for baseline symptoms and gender.

Analyses were conducted using R Version 3.3.0 [37]. As this study was exploratory, we did not include missing data sensitivity analyses [36].

## 3. Results

### 3.1. Sample Characteristics at Baseline

Participants ranged from 20 to 73 years, with an average age of 45.2 years (Standard Deviation (SD) 11.0). There were 113 men and 108 women. The majority of the DAISI participants reported experiencing a traumatic event (any of SA, PA, combat/torture or natural disaster that resulted in the PTSS or PTSD) (71.8%, *n* = 158) with over a third reaching the Diagnostic and Statistical Manual of Mental Disorders (4th ed., DSM-IV) criteria for current PTSD (37.7%, 83/220). There were no gender differences in rates of trauma exposure (*p* = 0.75), number of traumatic events experienced (*p* = 0.44), nor PTSD diagnosis (0.58). In this sample: 78 (41.1%) reported neither *SA* nor *PA* exposure; 28 (14.7%) reported SA but No PA; 38 (20%) reported PA but No SA, and 46 (24.2%) reported both SA and PA exposure: 30 participants did not provide a response.

### 3.2. Sexual Assault Events and Psychological Symptoms at Baseline

Of the 74 participants who had experienced SA, 33 reported one, 28 reported two, and 13 reported all three types of SA (Table 1). The observed proportion of females that had experienced SA was higher than males (57/94 = 60.6% vs. 17/96 = 17.7%) with a Chi-square test suggesting SA was more likely in women, χ^2^ (1) = 36.8, *p* < 0.001. Women were also more likely to have had multiple SA event types, χ^2^ (1) = 37.5, *p* < 0.001. The observed proportion of participants with PTSD was higher in the SA group than in the No SA group (58.1% vs. 34.5%) and a Chi-square test suggested a dependency between the level of trauma and SA status, χ^2^ (2) = 23.1, *p* < 0.001. The SA group also had more severe PTSS (difference between means = 5.1, 95% Confidence Interval (CI) 1.2 to 9.0, *p* = 0.01), depressive symptoms (4.0, 95% CI 1.4 to 6.7, *p* < 0.01), and alcohol dependence symptoms (3.7, 95% CI 0.2 to 7.1, *p* = 0.04) than the No SA group. As the number of SA types experienced increased, the more severe was the reported PTSS, with a statistically significant difference between groups at the *p* < 0.001 level. Similarly, depressive symptoms increased monotonically with the number of SA types experienced (*p* = 0.001) as did alcohol misuse (*p* = 0.05), and global functioning worsened (*p* = 0.001).

### 3.3. Physical Assault Events and Psychological Symptoms at Baseline

Of the 84 participants who had experienced PA, 54 reported one, 21 reported two, and 9 reported three types of PA (Table 2). PA participants (*n* = 84) were significantly younger than those who were in the No PA group (3.5 years, 95% CI 0.41 to 6.70, *p* < 0.05). The proportion of participants who were currently prescribed antidepressant medication decreased as the PA types experienced by a participant increased, with a statistically significant difference between groups (χ^2^ (3) = 8.4, *p* < 0.05). A significant association was also found between gender and the number of PA types experienced (*p* < 0.01 from Fisher Exact) with only men reporting having experienced more than 2 PA types. As the number of PA types experienced increased, the more severe the reported PTSS with a statistically significant difference between groups (*p* < 0.05). The PA group had more severe depressive symptoms (2.7, 95% CI 0 to 5.3, *p* = 0.05), lower global functioning (−4.8, 95% CI −7.8 to −1.8, *p* < 0.01), and worse alcohol-related measures than the No PA group. The depression symptoms increased monotonically with the number of PA types experienced (*p* < 0.05) as did all alcohol-related measures, and global functioning decreased (*p* = 0.006).

### 3.4. Treatment Allocation, Retention, and Follow-up Attrition

Of the 190 participants providing responses at baseline, 44 (23.2%) received the brief intervention; 45 (23.7%) received treatment for depression; 46 (24.2%) received treatment for alcohol; and 55 (29.0%) received the integrated treatment (refer Table 3). A chi-Square test of independence suggested no statistically significant dependence at the 0.05 level between SA/No SA group membership and treatment allocation (χ^2^ (3) = 0.72, *p* = 0.87). Similarly, no dependence was apparent between PA/No PA group membership and treatment allocation (χ^2^ (3) = 0.74 *p* = 0.87). Across all treatments there were 157, 137, 114, and 106 responses at the 3, 12, 24, and 36-month follow-ups respectively.

#### 3.4.1. SA Group

No differences were found in the number of treatment sessions attended by participants in the SA group (mean = 4.6, SD = 4.2) compared with those in the No SA group (M = 4.9, SD = 4.1), *p* = 0.54 (Table 1).

We found no association between SA status and the follow-up period (χ^2^ (4) = 0.4, *p* = 0.98). Furthermore, discrete-time survival analysis suggested that the number of follow-up periods was not significantly different between groups (hazard ratio = 1.11, 95% CI 0.71 to 1.71, *p* = 0.64).

#### 3.4.2. PA Group

The PA group attended fewer treatment sessions (*M* = 4.1, SD = 4.0) compared with those with No PA exposure (5.3 sessions, SD = 4.1), *p* < 0.05 (see Table 2).

We found no significant association between PA status and follow-up periods (χ^2^ (4) = 0.6, *p* = 0.97). Discrete-time survival analysis suggested that the number of follow-up periods was not significantly different between groups (hazard ratio = 1.07, 95% CI 0.69 to 1.64, *p* = 0.76).

### 3.5. Treatment and Follow-up Outcomes

The observed mean differences between the baseline and assessment time point in the depression score, global functioning, weekly and binge drinking, alcohol misuse and dependence symptoms and the model results, are shown in Table 4. The *p*-values in Table 4 correspond to the estimated difference between the group means at each follow-up period.

#### 3.5.1. Sexual Assault Group

The SA group had similar mean changes in symptom severity over the five follow-up periods to those in the No SA group, for all treatment outcome variables. The only exception was at the 12-month follow-up assessment where the SA group had significantly lower change scores (i.e., less improvement) in global functioning compared with those with No SA (modelled difference = −4.5, 95% CI −8.6 to −0.4, *p* < 0.05).

#### 3.5.2. Physical Assault Group

The PA group also had similar mean changes in symptom severity to those in the No PA group for all treatment outcome variables, over the five follow-up periods. At the 12-month follow-up, the PA group showed less improvement in global functioning (modelled difference = −5.9, 95% CI −9.8 to −2.1, *p* < 0.01) and alcohol dependence symptoms (modelled difference = 3.5, 95% CI 0.6 to 6.4, *p* < 0.05) compared with No PA.

### 3.6. Supplementary Analysis

Table 5 shows the observed mean differences from baseline in depression, global functioning, and alcohol dependence symptoms for each of the SA and PA, No SA and No PA, and SA and No PA groupings, and the model results. The *p*-values in Table 5 correspond to the modelled difference between the group means at each follow-up period. For depression, at the 12-month follow-up there was evidence of less improvement in the SA and PA group compared with the No SA and No PA group (5.3, 95% CI 0.1 to 10.5, *p* < 0.05), and in the SA and PA group versus the SA and No PA group (modelled difference = 7.0, 95% CI 0.6 to 13.4, *p* < 0.05). For global functioning at the 12-month follow-up, the SA and PA group showed less improvement than the No SA and No PA group (−9.3, 95% CI −14.3 to −4.2, *p* < 0.001). Similarly, SA and PA group showed evidence of less improvement in global functioning than both the versus the SA and No PA group (modelled difference = −8.2, 95% CI −14. 6 to −1.9, *p* < 0.05) and the No SA and *PA* group (−5.4463, 95% CI −11.1579 to 0.27 *p* = 0.06).

## 4. Discussion

This study found that participants with a history of SA or *PA* reported more severe depression, alcohol misuse, and poorer levels of global functioning at baseline, compared with those with no assault exposure. Although participants reporting past assaults responded well to our non-PTSD focused CBT at posttreatment, they had poorer symptom outcomes at the 12-month follow-up period. These findings have important clinical implications in the delivery of psychological therapy for coexisting depression and alcohol misuse among people with a history of SA or PA. One such clinical implication is the screening for assaults in depressed and alcohol misuse treatment seekers due to the initial severity of symptoms and possible deterioration by the 12-month follow-up.

The first hypothesis predicted that participants reporting past SA or PA would exhibit poorer attendance at treatment and follow-up. Whilst the SA group attended (on average) the same number of treatment sessions as the No SA group, the PA group attended fewer treatment sessions than the No PA group. Lower attendance rates were particularly reported by those with two or more PA event types (two sessions compared to five attended by the SA, No SA, and No PA groups). This suggests that participants with PA experiences may be more difficult to retain in treatment compared with those who experience SA.

We found negligible evidence for the second hypothesis that participants reporting assault exposure would have poorer treatment outcomes compared with participants with no assault exposure. Both SA and PA groups showed similar levels of symptom improvement over time, compared with those who were in the No SA and No PA groups. The only area of comparatively poorer functioning for the *SA* group was for global functioning at the 12-month follow-up period. PA participants were also more likely to experience poorer symptom outcomes at 12-months for global functioning and alcohol dependence symptoms. The supplementary analysis, however, found that SA and PA only participants had significantly more severe depressive symptoms and lower global functioning scores at the 12-month follow-up. This finding is different to the primary analysis that found the PA group had more severe alcohol dependence symptoms at the 12-month follow-up, which may be due to the smaller sample size in the supplementary analysis grouping. Overall, these results show that aftercare is required for both SA and PA to help maintain symptom improvement over time.

The similar rates of treatment success for the SA group (versus No SA) may be due to these participants being predominantly female. Research has found that compared with males, females are more likely to respond well to treatment [38]. This gender pattern has also been found to occur for CBT interventions in substance using populations [39]. Other studies have also found that sexually assaulted females respond well to integrated CBT for depression [40,41].

The comparatively poor symptoms for the PA (versus No PA) group in global functioning and severity of alcohol dependence symptoms at the 12-month follow-up may have been influenced by treatment and session participation, as this group spent significantly fewer days in treatment. Poorer comorbid symptom outcome for the PA (versus No PA) group may also have been influenced by participants (who reported two or more PA event types) not taking antidepressant medication. The poorer outcomes for the PA (versus No PA) group at the 12-month assessment suggests that the *PA* participants may require additional (or booster) treatment sessions 12 months post-treatment to maintain symptom improvements.

The maintained improvement in most of the treatment outcome variables over most of the follow-up periods may have also been the result of the CBT model being the basis of the DAISI interventions. High quality CBT programs have been found to have greater effects for participants at high risk of relapse [42]. CBT may also be better suited for longer-term outcomes [39,43] as the skills that are taught appear to be retained after treatment [44].

The main limitation of this study was that the parent study recruited participants with depressive symptoms and alcohol misuse rather than an assault exposure population, affecting assault allocation and obtaining further information about assault history. This recruitment limitation also resulted in limited power to determine if there were any between-group differences for SA versus No SA and PA versus No PA by the four treatment conditions. Also, the long-term effects of treatment on PTSS could not be determined as the parent study did not measure this variable beyond 3 months. We would suggest caution with interpreting our results due to the number of tests conducted. Even though these issues raised are research limitations, these findings may be reflective of what occurs in mental health, and alcohol and other drug treatment services, whereby patients often present for treatment of other symptoms (as per DAISI project) [6].

## 5. Conclusions

This study showed that people who have coexisting depressive symptoms and alcohol misuse, and who have been assaulted, can respond well to non-PTSD focused CBT, and that improvement is maintained over time. However, people who report PA may be more difficult to retain in treatment compared with those who have not experienced PA. Furthermore, people who have been assaulted may also experience more severe symptoms and comparatively poorer global functioning 12 months post treatment. Therefore, it may be beneficial for health care workers to screen for traumatic events, monitor outcomes over time, and offer booster sessions to comorbid populations who experienced assaults.

## Figures and Tables

**Table 1:** Demographic characteristics at the baseline assessment for SA (and number of SA event types) compared with No SA.

Demographic Characteristics	Between Groups			Number of Sexual Assault Event Types				Statistics
	SA *n* = 74	No SA *n* = 116	Statistics	0 *n* = 116	1 *n* = 33	2 *n* = 28	3 *n* = 13	
	*n* (%)	*n* (%)	*p*-value	*n* (%)	*n* (%)	*n* (%)	*n* (%)	*p*-value
Gender								
Males	17 (17.7)	79 (82.3)	<0.001	79 (82.3)	6 (6.2)	8 (8.3)	3 (3.1)	<0.001
Females	57 (60.6)	37 (39.4)		37 (39.4)	27 (28.7)	20 (21.3)	10 (10.6)	-
Antidepressant	42 (42.4)	57 (57.6)	0.36	57 (57.6)	20 (20.2)	15 (15.2)	7 (7.1)	0.64
	Mean (SD)	Mean (SD)		Mean (SD)	Mean (SD)	Mean (SD)	Mean (SD)	
Age	43.5 (11.0)	46.1 (11.0)	0.11	46.1 (11.0)	42.7 (9.8)	45.0 (13.4)	42.1 (8.3)	0.34
Treatment								
Sessions Attended	4.6 (4.2)	4.9 (4.1)	0.54	4.9 (4.1)	4.6 (4.3)	4.5 (4.2)	4.6 (4.3)	0.94
Days in Treatment	45.8 (50.7)	52.5 (52.9)	0.39	52.5 (52.9)	45.5 (52.6)	45.8 (49.5)	46.7 (52.4)	0.87
Symptoms								
PTSS	23.1 (13.0)	18.0 (11.8)	<0.05	18.0 (11.8)	18.8 (9.2)	23.8 (14.1)	32.4 (14.6)	<0.001
Depression	34.4 (9.9)	30.3 (8.7)	<0.01	30.3 (8.7)	31.5 (8.4)	35.4 (10.5)	39.5 (10.5)	<0.001
Global Functioning	54.5 (12.1)	57.4 (8.8)	0.06	57.4 (8.8)	59.0 (10.6)	52.0 (11.5)	48.5 (13.7)	<0.01
Alcohol								
TLFB Weekly Drinking	61.2 (42.5)	66.3 (47.2)	0.46	66.3 (47.2)	55.6 (42.3)	66.6 (45.8)	62.9 (36.0)	0.70
TLFB Binge Drinking	18.0 (9.6)	17.6 (10.3)	0.817	17.6 (10.3)	16.8 (7.1)	17.1 (10.4)	23.2 (12.3)	0.27
Alcohol Misuse	27.2 (6.8)	25.3 (6.7)	0.07	25.3 (6.7)	26.9 (5.8)	25.9 (7.8)	30.5 (5.8)	0.05
Alcohol Dependence Symptoms	20.1 (12.1)	16.4 (11.1)	<0.05	16.4 (11.1)	18.8 (9.8)	20.7 (14.9)	22.5 (12.0)	0.16

Note: PTSS = Posttraumatic Stress Symptoms; TLFB = Time Line Follow-Back; SA = Sexual Assault; PA = Physical Assault, SD = Standard Deviation.

**Table 2:** Demographic characteristics at the baseline assessment for PA (and number of PA event types) compared with No PA.

Demographic Characteristics	Between Groups			Number of Physical Assault Event Types				Statistics
	PA *n* = 84	No PA *n* = 106	Statistic	0 *n* = 106	1 *n* = 54	2 *n* = 21	3 *n* = 9	
	*n* (%)	*n* (%)	*p*-value	*n* (%)	*n* (%)	*n* (%)	*n* (%)	*p*-value
Gender								
Males	41 (42.7)	55 (57.3)	0.78	55 (57.3)	23 (24.0)	9 ( 9.4)	9 (9.4)	<0.01
Females	43 (45.7)	51 (54.3)	-	51 (54.3)	31 (33.0)	12 (12.8)	0 (0.0)	
Antidepressant	37 (37.4)	62 (62.6)	0.09	62 (62.6)	25 (25.3)	11 (11.1)	1 (1.0)	<0.05
	Mean (SD)	Mean (SD)		Mean (SD)	Mean (SD)	Mean (SD)	Mean (SD)	
Age	43.1 (10.9)	46.6 (10.9)	<0.05	46.6 (10.9)	44.4 (10.6)	40.8 (12.0)	40.6 (10.6)	0.07
Treatment								
Sessions Attended	4.1 (4.0)	5.3 (4.1)	<0.05	5.3 (4.1)	5.1 (4.0)	2.1 (2.9)	2.4 (4.3)	<0.01
Days in Treatment	42.6 (49.0)	55.6 (53.9)	0.09	55.6 (53.9)	54.6 (49.9)	18.8 (33.9)	26.4 (52.4)	<0.05
Psychological Symptoms								
Posttraumatic Stress Symptoms	21.7 (13.7)	19.0 (11.2)	0.18	19.0 (11.2)	18.8 (13.2)	25.0 (14.7)	30.3 (9.2)	<0.05
Depression	33.4 (10.2)	30.7 (8.5)	0.05	30.7 (8.5)	31.9 (10.3)	34.7 (9.8)	39.1 (8.5)	<0.05
Global Functioning	53.6 (11.7)	58.4 (8.4)	<0.01	58.4 (8.4)	54.9 (11.1)	51.7 (14.3)	50.3 (9.4)	<0.01
Alcohol								
TLFB Weekly Drinking	75.1 (51.2)	55.1 (37.5)	<0.01	55.1 (37.5)	65.0 (40.9)	78.3 (58.7)	134.2 (57.6)	<0.001
TLFB Binge Drinking	20.3 (11.1)	15.7 (8.4)	<0.01	15.7 (8.4)	17.3 (8.2)	22.7 (14.1)	33.3 (9.5)	<0.001
Alcohol misuse	27.5 (6.7)	24.9 (6.6)	<0.01	24.9 (6.6)	26.6 (6.3)	28.7 (7.3)	30.2 (6.6)	<0.05
Alcohol Dependence Symptoms	20.1 (12.2)	16.0 (10.7)	<0.05	16.0 (10.7)	16.5 (10.1)	27.1 (14.0)	28.9 (11.5)	<0.001

**Table 3:** Sample size and percentage for assault groups by treatment allocation.

Assault Grop Sample Size	Treatment Allocation			
	Brief Intervention (90 min)	Depression (10 Sessions)	Alcohol (10 Sessions)	Integrated (10 Sessions)
	*n* (%)	*n* (%)	*n* (%)	*n* (%)
Total Assault Sample (*n* = 220)	44 (23.2%)	45 (23.7%)	46 (24.2%)	55 (29.0%)
Assault Group				
SA (*n* = 74)	18 (24.3%)	19 (25.7%)	18 (24.3%)	19 (25.7%)
No SA (*n* = 116)	26 (22.4%)	26 (22.4%)	28 (24.1%)	36 (31.0%)
PA (*n* = 84)	21 (25.0%)	21 25.0(%)	20 (23.8%)	22 (26.2%)
No PA (*n* = 106)	23 (21.7%)	24 (22.6%)	26 (24.5%)	33 (31.1%)

Note: 30 did not provide SA or PA data; Total Assault sample percentages for treatment conditions are reported horizontally. Assault Group percentages within treatment conditions are reported vertically.

**Table 4:** Treatment outcomes (mean change scores) for SA vs. No SA and PA vs. No PA, over the five assessment periods.

Assessment Period	SA *n* = 74	No SA *n* = 116	Statistics	PA *n* = 84	No PA *n* = 106	Statistics
	Mean (SD)	Mean (SD)	*p*-Value	Mean (SD)	Mean (SD)	*p*-Value
Treatment Outcomes						
Depression						
3 months	−12.4 (12.6)	−11.2 (12.2)	0.75	−10.4 (12.1)	−12.6 (12.5)	0.08
6 months	−12.9 (15.4)	−11.4 (12.1)	0.76	−12.6 (13.4)	−11.4 (13.4)	0.81
12 months	−13.7 (14.7)	−13.9 (10.5)	0.29	−12.4 (13.3)	−15.0 (11.1)	0.08
24 months	−14.0 (13.4)	−13.7 (9.9)	0.72	−15.4 (12.3)	−12.7 (10.3)	0.84
36 months	−16.5 (12.9)	−14.2 (12.9)	0.86	−16.3 (13.1)	−14.2 (12.8)	0.88
Global Functioning						
3 months	7.3 (13.2)	8.0 (11.8)	0.47	7.8 (13.5)	7.7 (11.4)	0.25
6 months	8.4 (13.6)	6.1 (12.3)	0.83	7.4 (13.6)	6.5 (12.0)	0.28
12 months	6.8 (13.5)	9.7 (11.1)	<0.05	6.4 (12.2)	10.4 (11.7)	<0.01
24 months	13.2 (15.1)	9.4 (10.0)	0.15	13.0 (14.9)	9.1 (9.4)	0.36
36 months	12.8 (15.0)	12.9 (9.8)	0.44	14.5 (12.5)	11.6 (11.5)	0.70
TLFB Weekly Drinking						
3 months	−22.0 (34.5)	−22.0 (45.0)	0.92	−19.9 (45.1)	−23.7 (37.6)	0.15
6 months	−25.5 (41.2)	−26.2 (41.7)	0.88	−28.6 (45.8)	−23.7 (37.2)	0.29
12 months	−22.0 (38.6)	−21.8 (47.6)	0.88	−26.5 (53.2)	−17.9 (35.0)	0.76
24 months	−31.7 (29.2)	−15.7 (52.7)	0.06	−26.5 (42.2)	−17.3 (49.2)	0.99
36 months	−22.7 (43.8)	−26.6 (45.8)	0.92	−27.3 (44.7)	−23.3 (45.4)	0.70
TLFB Binge Drinking						
3 months	−7.6 (10.5)	−5.4 (11.0)	0.98	−6.8 (10.9)	−5.9 (10.8)	0.29
6 months	−7.3 (10.1)	−6.6 (11.2)	0.41	−8.0 (11.7)	−5.8 (9.8)	0.44
12 months	−8.9 (9.6)	−6.3 (10.6)	0.96	−9.0 (10.1)	−5.8 (10.2)	0.91
24 months	−9.6 (12.1)	−5.3 (10.0)	0.51	−8.5 (12.6)	−5.6 (9.3)	0.66
36 months	−8.5 (8.6)	−6.1 (10.9)	0.96	−7.9 (10.5)	−6.3 (9.7)	0.96
Alcohol Misuse						
3 months	NA	NA	-	NA	NA	-
6 months	−5.0 (9.7)	−5.0 (7.5)	0.89	−5.3 (8.8)	−4.8 (8.0)	0.85
12 months	−5.3 (7.3)	−6.4 (7.8)	0.50	−5.8 (8.1)	−6.1 (7.2)	0.45
24 months	−6.8 (14.7)	−7.2 (7.5)	0.949	−7.8 (14.0)	−6.5 (7.4)	0.87
36 months	−9.2 (9.3)	−7.2 (7.7)	0.45	−10.4 (8.7)	−6.3 (7.8)	0.21
Alcohol Dependence Symptoms						
3 months	NA	NA	-	NA	NA	-
6 months	−1.0 (7.7)	0.8 (8.9)	0.69	0.4 (8.7)	0.0 (8.5)	0.34
12 months	−1.2 (6.8)	−0.6 (8.1)	0.630	0.6 (8.8)	−1.8 (6.6)	<0.05
24 months	−2.3 (8.8)	−1.5 (8.0)	0.89	−1.5 (9.1)	−1.9 (7.9)	0.18
36 months	−3.2 (6.9)	−0.2 (8.9)	0.61	−1.3 (8.3)	−1.3 (8.3)	0.49

Note: *n* for assault groups at baseline.

**Table 5:** Treatment outcomes (mean change scores) for “SA and PA” vs “No SA and No PA” vs “SA and No PA” vs “No SA and PA”, over the assessment periods.

Assessment Period	SA, PA *n* = 46	No SA, No PA *n* = 78	Statistics	SA, No PA *n* = 28	Statistics	No SA, PA *n* = 38	Statistics
	Mean (SD)	Mean (SD)	*p*	Mean (SD)	*p*	Mean (SD)	*p*
Treatment Outcomes							
Depression							
3 months	−10.7 (11.5)	−11.8 (11.9)	0.17	−14.7 (13.8)	0.09	−10.2 (12.9)	0.66
6 months	−12.1 (14.2)	−10.5 (11.8)	0.53	−14 (17.4)	0.19	−13 (12.8)	0.27
12 months	−11.9 (14.6)	−14.5 (9.7)	<0.05	−16.6 (14.6)	<0.05	−12.9 (12)	0.15
24 months	−14.6 (14.1)	−12.4 (9.3)	0.80	−13.4 (12.9)	0.53	−16.1 (10.7)	0.33
36 months	−16.3 (13.2)	−13 (12.7)	0.85	−16.8 (12.9)	0.54	−16.3 (13.3)	0.75
Global Functioning							
3 months	8.1 (14.2)	8.3 (11.4)	0.18	6 (11.8)	0.46	7.5 (12.9)	0.63
6 months	9.5 (14.1)	6.5 (11.9)	0.49	6.5 (12.7)	0.41	5.4 (13)	0.66
12 months	5.3 (15.1)	10.8 (12.1)	<0.001	9.2 (10.6)	<0.05	7.5 (8.4)	0.06
24 months	17 (16.8)	9.6 (9.0)	0.13	7.9 (10.7)	0.35	9.1 (11.9)	0.15
36 months	16.2 (14.7)	12.9 (9.8)	0.80	8.6 (14.7)	0.42	12.9 (10.2)	0.95
Alcohol Dependence Symptoms							
6 months	−0.8 (8.7)	0.4 (9.1)	0.89	−1.4 (6.3)	0.63	1.3 (8.7)	0.63
12 months	−1.2 (7.2)	−2 (6.7)	0.17	−1.2 (6.4)	0.59	2.3 (10)	0.43
24 months	−2.3 (9.4)	−1.8 (7.8)	0.40	−2.2 (8.4)	0.47	−0.5 (8.9)	0.99
36 months	−3.7 (7.4)	−0.4 (9.1)	0.94	−2.8 (6.8)	0.61	0.3 (8.8)	0.74
SA = Sexual Assault; PA = Physical Assault, SD = Standard Deviation.							
